# Mental disorders management in general practice

**DOI:** 10.1192/j.eurpsy.2021.1065

**Published:** 2021-08-13

**Authors:** M. Turki, T. Babbah, W. Abid, S. Ellouze, R. Ouali, R. Charfi, N. Halouani, J. Alouou

**Affiliations:** Psychiatry “b” Department, Hedi Chaker University hospital, sfax, Tunisia

**Keywords:** Psychiatric disorders, management, perception, general practice

## Abstract

**Introduction:**

Compared to specialized care, primary care is considered to be more accessible, less stigmatizing, and more comprehensive since it manages physical ailments along with mental disorders (MD). Thus, MD are mainly treated by general practitioners (GP), even though their ability to diagnose and treat these diseases is often considered unsatisfactory.

**Objectives:**

This study aimed to analyze perceptions of GP capacity to manage MD, and to assess the difficulties encountered during this management.

**Methods:**

A cross-sectional web-based survey design was adopted between August 22 and September 23, 2020, so that 47 responses of GP were included.

**Results:**

The mean age of respondents was 37.3 years. Among them, only 17% attended a post-university psychiatric training. On average, 6.3% of GP visits were MD-related. Anxious disorders and depression were perceived as very frequent respectively in 82.9% and 40.4% of cases. Among GP, 17% considered bipolar disorder as a difficult pathology to diagnose, followed by schizophrenia (12.7%), while the pathologies perceived to be most difficult to treat were dementia (17%), acute agitations (14.9%) and schizophrenia (10.6%). Anxiolytics and antidepressants use was very frequent (40.4% and 27.7% respectively), and 34% needed training in antipsychotics prescription. Difficulties encountered during MD management were related to lack of psychiatric continuing education (19.4%) and lack of collaboration with mental health professionals (12.5%). Among participants, 93.6% requested a psychiatric training: theoretical 29.3%, practice exchange 24.7%.

**Conclusions:**

Our study confirmed that MD related visits are common in primary care and highlighted several obstacles in their management. Further continuous education, training,and collaboration between practitioners is required.

